# Synthesis and Functions of Resistant Starch

**DOI:** 10.1016/j.advnut.2023.06.001

**Published:** 2023-06-03

**Authors:** Zhanggui Wang, Shuli Wang, Qinhong Xu, Qi Kong, Fei Li, Lin Lu, Yibiao Xu, Yali Wei

**Affiliations:** 1 Department of Radiotherapy, Anhui No. 2 Provincial People’s Hospital, Hefei, China; 2 Department of Women’s Health, Jiaxing Maternity and Child Health Care Hospital, Affiliated Women and Children's Hospital of Jiaxing University, Jiaxing, China; 3 Department of Radiology, Shandong Provincial Hospital Affiliated to Shandong First Medical University, Jinan, China; 4 Department of Acupuncture and Massage, Anhui No.2 Provincial People’s Hospital, Hefei, China; 5 Department of Neurosurgery, The Fifth People's Hospital of Huai 'an, Huai' an, China

**Keywords:** resistant starch, digestion, preparation, function, intestinal flora

## Abstract

Resistant starch (RS) has become a popular topic of research in recent years. Most scholars believe that there are 5 types of RS. However, accumulating evidence indicates that in addition to starch–lipid complexes, which are the fifth type of RS, complexes containing starch and other substances can also be generated. The physicochemical properties and physiologic functions of these complexes are worth exploring. New physiologic functions of several original RSs are constantly being discovered. Research shows that RS can provide health improvements in many patients with chronic diseases, including diabetes and obesity, and even has potential benefits for kidney disease and colorectal cancer. Moreover, RS can alter the short-chain fatty acids and microorganisms in the gut, positively regulating the body’s internal environment. Despite the increase in its market demand, RS production remains limited. Upscaling RS production is thus an urgent requirement. This paper provides detailed insights into the classification, synthesis, and efficacy of RS, serving as a starting point for the future development and applications of RS based on the current status quo.

**Statement of Significance**

This paper provides detailed insights into the classification, synthesis, and efficacy of resistant starch, guiding the future development and applications of resistant starch based on the current status quo.

## Introduction

Starch is a polymer composed of glucose subunits. Based on the type of polymerization, starch can be divided into 2 categories: amylose and amylopectin. Amylose has a linear structure consisting of D-glucose residues linked by α-1,4-glycosidic bonds and a molecular weight of ∼1 × 10^5^ to 1 × 10^6^. Further, each chain of amylose contains ∼200 to 700 glucose residues. In contrast, amylopectin is a much larger molecule than amylose, with a molecular weight of 1 × 10^7^ to 1 × 10^9^. Its degree of polymerization ranges from 9600 to 15,900 glucose units [1]. Amylopectin is connected by α-1,4-glycosidic bonds and α-1,6-glycosidic bonds. Amylose and amylopectin coexist in nature. However, according to the proportion of amylose and amylopectin, starch can be subdivided into the following 3 types: “waxy” starch, “normal” starch, and “high-amylose” starch. In “waxy” starch, amylopectin is the predominant molecule, accounting for ∼98% to 99% of all starch molecules. “Normal” starch contains ∼25% to 30% amylose, whereas amylose accounts for >50% of the starch molecules in “high-amylose” starch.

As a carbohydrate, starch is a key source of energy for physiologic processes in the human body. Previously, it was believed that all starches can be enzymatically digested in the digestive system, before finally being absorbed into circulation. However, with additional research, a unique type of starch that cannot be digested and absorbed by the small intestine was discovered [[2], [3], [4]]. In 1982, Englyst et al. [2] first named this starch “resistant starch” (RS). In 1991, European Concerted Action confirmed that RS is not digested and absorbed in the small intestine. Instead, it reaches the colon, where it is fermented to variable degrees by gut microbes [5]. In 1992, The FAO of the United Nations deemed RS to be the general term for starches and starch degradation products that cannot be digested by the small intestine in healthy people [6].

In addition to the naturally occurring RS that was originally discovered by Englyst, different types of RS have been synthesized through the artificial modification of starch. These novel RSs have shown better performance than natural RS. The increase in the demand for RS has bolstered research on RS synthesis, with scientists hoping to mass-produce RS to meet consumer needs. As a natural ingredient, RS can have broad applications in the food and health industries. RS can be used to improve food processing given its low water-holding capacity and fine texture-related organoleptic properties [7]. It also increases crispness and adds bulk to foods, improving the texture of the final product [8]. Hence, RS can be incorporated into different types of functional and flavored foods. Moreover, RS has clinical applications because it is not digested in the small intestine. Thus, it can be used as the wall material of microcapsules to induce drug release at specific sites in the body [9]. As an additive, RS can effectively control body weight and delay blood sugar spikes while providing other physiologic benefits [10,11]. Accordingly, it can be used to develop foods for special medical purposes. RS can provide certain benefits for the prevention and treatment of chronic diseases, such as diabetes, obesity, and hyperlipidemia. The effects of RS on health are being explored through animal as well as human studies, and its physiologic mechanisms are being investigated at the molecular level in cell models [12,13].

In this review, we describe the recent classification of RS, its in vivo digestion, the current progress in RS synthesis methods, and the physiologic functions of RS in the human body. This summary of the latest findings on RS could inspire the practical application of RS.

## Classification of RS

### Basic classification

When Englyst et al. [14] first proposed the concept of RS, RS was divided into 3 types: RS_1_, RS_2_, and RS_3_. Natural starch granules are usually encapsulated within plant components (such as the cell wall or proteins), forming a physically embedded starch called RS_1_. This encapsulated structure hinders contact between amylase and starch within the digestive system, resulting in enzymatic protection. RS_1_ is mainly found in natural grains and beans that have not been ground sufficiently. In contrast, RS_2_ is a natural raw starch granule with a special crystalline structure and high-starch density. This special property reduces its sensitivity to enzymes. Therefore, RS_2_ can resist the hydrolysis caused by digestive enzymes to a certain extent. Raw potatoes and bananas have a high content of RS_2_. However, this type of starch is easily destroyed by heat. When starch is heated to a temperature higher than its gelatinization temperature, its original crystal structure is destroyed. When the gelatinized starch is placed at a low temperature for a certain period of time, it undergoes a retrogradation process to produce retrograded starch, namely RS_3_.

Following in-depth research on RS, Englyst et al. [15] identified RS_4_—a class of chemically modified starches. RS_4_ is resistant to digestive enzymes because its original functional groups have been chemically modified or new functional groups have been introduced to produce carboxymethyl starch, starch ether, starch ester, and cross-linked starch [16]. In addition, the long branches of amylose or amylopectin have been combined with FAs to generate a starch–FA complex that cannot be penetrated by water or amylase. This type of starch has also been classified as an RS and named RS_5_ [17].

During food processing, starch often interacts with other ingredients. In addition to lipids, other nutrients, such as proteins, are also present. Several studies have demonstrated the formation of ternary starch–lipid–protein complexes that affect starch digestibility during processing [[18], [19], [20], [21]]. It remains to be confirmed whether these starch–lipid–protein complexes share the same properties as other types of RS, including their physiologic function. In some cases, proteins play a positive role in the complexation of starch and lipids [[21], [22], [23]]. The definition of RS is based on its resistance to digestive enzymes. Hence, starch–lipid–protein complexes should also be considered a type of RS if they can resist digestive enzymes and produce health-promoting effects upon entering the digestive system. The purpose of RS classification is to explore the beneficial physicochemical properties and functions of RS. Through in-depth research on starches, deeper insights into the definition and classification of RS can be obtained in the future.

### Dietary fiber and RS

Due to its complex molecular structure and different functions, the definition of dietary fiber has been debated for many years. According to most definitions, dietary fiber includes 4 major substances: resistant oligosaccharides, nonstarch polysaccharides, RS, and noncarbohydrate compounds [24]. In terms of physiologic functions, dietary fiber has several effects: *1*) improving blood glucose and insulin levels, *2*) reducing blood lipid levels, and *3*) improving defecation [[25], [26], [27], [28], [29], [30]]. The European Food Safety Authority classified dietary fiber as “soluble dietary fiber” and “insoluble dietary fiber” based on its physicochemic properties and physiologic functions [31,32]. Traditionally, soluble dietary fiber is believed to promote defecation and increase fecal weight, whereas insoluble dietary fiber is beneficial for reducing blood lipid concentrations. The health effects of RS, such as improved glucose and lipid metabolism and a reduced risk of colorectal cancer, are closely related to these properties of soluble dietary fiber. From a classification point of view, RS is largely considered a dietary fiber. After the initial discovery of RS, in vitro and in vivo experiments were performed to examine whether its physiologic functions were consistent with the properties of dietary fiber. However, with progress in research, new types of RS were discovered, and nonstarch substances were introduced into its molecular structure. These modifications altered the rate of RS-induced fermentation in vivo and the microenvironmental changes it causes in the gut [33]. As a heterogeneous fiber subgroup, RS has good functional properties in food products, especially given its low water-holding capacity, and it has more application prospects than traditional dietary fibers [34]. Thus, further studies in human subjects are warranted because previous research has yielded conflicting results [35], with functional differences between RS and other dietary fibers and between different types of RS as well.

### Prebiotics and RS

The definition of prebiotics has continuously been updated since it was first put forth by Gibson and Roberfroid [36] in 1995. In 2017, the International Scientific Association of Probiotics and Prebiotics defined prebiotics as follows: “*a substrate that is selectively utilized by host microorganisms, conferring a health benefit*” [37]. In recent decades, studies have shown that prebiotics not only produce beneficial gastrointestinal effects, but also have positive hematologic, cardiovascular, and cognitive effects [[38], [39], [40]]. The latest evidence shows that prebiotics may also play an immunomodulatory role in preventing COVID-19 [41,42]. In addition to prebiotics, such as the oligosaccharides fructans and galactans, several new molecules with prebiotic effects are gradually being uncovered. One example of these new molecules is RS. Notably, RS has been shown to have prebiotic potential in regulating intestinal microorganisms [43].

Compared with its dietary fiber-related properties, the prebiotic properties of RS are more challenging to observe. There is a great difference between the results obtained in vitro, in animal studies in vivo, and in clinical trials. To study the prebiotic properties of RS, carefully designed human studies are required. The fifth part of this review collates all current population-based randomized controlled trials on RS and its effects on the gut microbiota. These studies focus on RS_2_, RS_3_, and RS_4_. The results show that the effects of RS differ among different microbial species, even within the same colony. However, RS generally increases the number of bacteria beneficial to the human body. Therefore, to understand whether RS can be converted into a prebiotic, different types of RS need to be studied.

Overall, dietary fiber, RS, and prebiotics appear to share some overlap. Although RS is a dietary fiber, all dietary fibers are not prebiotics, and all types of RS are also not prebiotics.

### Digestion of RS

The 4 kinds of traditional RSs resist decomposition by digestive enzymes in the small intestine and enter the large intestine. The large intestine has an anaerobic environment and various microorganisms, including beneficial bacteria.

### Digestion in the large intestine

RS interacts with gut microbes to produce SCFAs and some gases. SCFAs include acetic acid, butyric acid, and propionic acid, and the latter 2 account for ∼80% of all gut SCFA. Moreover, the gases include carbon dioxide, methane, hydrogen, and hydrogen sulfide. Gut microbes have different sensitivities to different types of RS [44,45]. Correspondingly, the types and amounts of SCFAs produced by different RSs in the intestines are different. These differences can explain the functional differences of RSs. Currently, the intestinal microorganisms known to effectively degrade RS in the colon are *Ruminococcus bromii* and *Bifidobacterium adolescentis* [46]. The metabolites produced by the bacterial fermentation of RS can serve as substrates for the growth of other microbial populations. This process is called cross-feeding. *Ruminococcus bromii* plays an important role in degrading RS to produce SCFA, promoting the reproduction of other beneficial bacteria through cross-feeding [47,48]. Although *Bifidobacterium adolescentis* and *Ruminococcus bromii* play a similar role, they release lactic acid and polysaccharides for cross-feeding through 2 different pathways, which can stimulate the production of butyrate by other intestinal bacteria [49].

### Hypothesized digestion of starch complex

It should be noted that the digestion of starch–lipid complexes differs from that of other RSs. Rice starch–oleic acid complexes are beneficial for the reproduction of butyrate-producing bacteria in rats [50]. This confirms that starch–lipid complexes are fermented in the large intestine, like other types of RSs, and can also cause changes in the gut microbiota. We speculate that the starch within these complexes is fermented, releasing FFA, which do not undergo oxidative modification due to the anaerobic environment of the large intestine [51]. It is possible that a hydrogenation reaction occurs, converting unsaturated FAs into SFAs [52]. Finally, FFAs are eliminated from the body. This hypothesis needs to be validated in future studies.

In the small intestine, the original structure of starch is destroyed by pancreatic α-amylase, resulting in the release of amylose. Lipids are digested by lipase to produce FFAs. In the neutral environment of the small intestine, amylose encounters FFAs, which likely promotes the formation of starch–lipid complexes. However, in the small intestine, starch is broken down into monosaccharides, and FFAs are reassembled into micelles [53]. These 2 processes compete with complex formation. The hypothesized formation of complexes in the small intestine remains to be validated. However, evidence from animal feed studies seems to support this view. The main components of animal feed are starch and fat. TGs constitute a large proportion of this fat, and TG metabolites contain FFAs. The formation of complexes should be minimized when the feed is digested in the animal’s body, allowing maximal energy yield and reducing the cost of feeding. Several animal experiments have proven that increasing the ratio of FFAs in the feed can reduce energy utilization, whereas increasing the ratio of unsaturated FAs promotes energy utilization [[54], [55], [56]]. In the small intestine, FFAs and amylose from the feed form starch–lipid complexes. In the large intestine, the starch in these complexes is fermented to produce SCFA. The FFAs remaining in the complexes are excreted from the body. The energy-generating nutrients in the feed cannot be digested and absorbed completely, resulting in energy loss. An in vitro digestibility study showed that starch–lipid–protein complexes are more resistant to amylase than starch–lipid complexes [21]. It is possible that the combination of 3 or even more compounds generates greater resistance to enzymatic hydrolysis, which remains to be studied.

### Measurement methods

In vitro experiments to measure the content of RS have largely been performed using 2 main methods: the direct method and the indirect method. Direct measurement is performed by converting RS into glucose [4]. First, digestive enzymes are added to digest the non-RS. Then, the RS residue is dissolved in potassium hydroxide or DMSO, and the glucose content is calculated after starch glucosidase conversion. The direct detection method has been continuously optimized. Goñi was the first to add pepsin to the detection system to simulate the human gastrointestinal tract [57]. The addition of pepsin allows protein removal, increasing the accessibility of starch granules for amylase while preventing starch–protein binding and the formation of protein-based starch-embedded particles [58]. The principle of the indirect method is based on the subtraction of the portion that can be digested by amylase from the total weight. The remaining portion is considered RS [15]. Starch is divided into fast-digestion starch (starch that can be hydrolyzed by amylase within 20 min), slow-digestion starch (starch that can be hydrolyzed by amylase within 20–120 min), and RS (starch that is not hydrolyzed by amylase within 120 min) based on the time taken for its digestion in the human body. The total starch minus fast-digestion starch and slow-digestion starch is considered the RS. This method of measuring digestibility for detecting RS content is helpful in the detection of new RSs. The direct method ignores other substances, resulting in the measured values being lower than the actual value. That is, the complexes do not only contain starch and lipid, but may also contain proteins, as is the case for ternary complexes. Regardless of which method is used, assays for the content of RS lay the foundation for the synthesis and functional evaluation of RS.

## Synthesis of RS

### Raw materials

Many factors affect the RS yield during the synthesis process. The most important of these factors is the raw material used. Table 1 [[59], [60], [61], [62], [63], [64], [65], [66], [67], [68], [69], [70], [71], [72], [73], [74], [75], [76], [77], [78], [79], [80], [81], [82], [83], [84], [85]] shows that RS can be prepared from different raw materials. The variation in the obtained RS content is relatively small when potato, sweet potato, and cassava are used as raw materials [60,[71], [72], [73], [74], [75], [76]]. However, the obtained RS content varies greatly when cereals like corn, wheat, and rice are used as raw materials [[59], [60], [61], [62], [63], [64], [65], [66], [67], [68]]. This difference is closely related to the chemical composition and physical structure of the starch present in these grains. Some Chinese medicinal plants, such as lotus seeds, yam, Gorgon, and Pueraria, are also used for the synthesis of RS [74,[79], [80], [81],[83], [84], [85]]. In addition, because of its high-starch content, *Canna edulis* has also been explored as a raw material for RS synthesis [82].

**TABLE 1:** Yield of resistant starch prepared using different raw materials and methods.

Material	Native	Methods	Type of resistant starch	Yield	Growth
High-amylose corn starch [59]	32.1%	Microwave irradiation treatment	RS_3_	43.4%	26.0%
Corn starch [60]	62.0%	Microwave irradiation retrogradation treatment	RS_3_	71.4%	15.2%
High-amylose corn starch [61]	/	Autoclaving–cooling treatment and acid hydrolysis	RS_3_	30.41%	/
Maize flour [62]	1.8%	Autoclaving–cooling and α-amylase treatment	RS_3_	14%	677.8%
Waxy corn starch [63]	13%	Pullulanase debranching treatment	RS_3_	19%	46.2%
Normal maize starch[64]	0.39%	Sonication and cross-linking treatment	RS_4_	75.9%	19,361.5%
Corn starch [65]	2.5%	Water bath treatment	RS_4_	78.4%	3036.0%
Normal corn starch [66]	13.2%	Autoclaving-acid treatment	Corn starch-palmitic acid complexes	25.8%	95.5%
Waxy corn starch [66]		Water bath treatment	Amylosucrase-modified waxy corn starch	43.5%	229.5%
Modified waxy corn starch [66]	43.5%	Autoclaving-acid treatment	Amylosucrase-modified waxy corn starch– myristic acid complexes	35.7%	−17.9%
Normal corn starch [67]	2.9%	Annealing treatment	RS_3_	8.2%	182.8%
			Corn starch–corn oil complexes	6.6%	127.6%
			Corn starch–soy protein complexes	9.5%	227.6%
			Corn starch–corn oil–soy protein complexes	5.9%	103.4%
Rice starch [68]	20.79%	Enzymatic hydrolysis	RS_3_	34.43%	65.6%
Wheat starch [65]	3.0%	Water bath treatment	RS_4_	95.8%	3093.3%
Buckwheat starch [69]	1.19%	Pressure-cooling cycles and pullulanase treatment	RS_3_	4.33%	263.9%
		Autoclaving–cooling and pullulanase treatment	RS_3_	5.87%	393.3%
High-amylose Tartary buckwheat starch [70]	31.58%	Physical mixing	High-amylose Tartary buckwheat starch flavonoid complex	33.65%	6.6%
		Water bath treatment		37.19%	17.8%
		Acid–base precipitation		36.45%	15.4%
		Microwave treatment		38.85%	23.0%
		Ultrasonic treatment		40.51%	28.3%
Yellow sweet potato [71]	24.1%	Heat–moisture treatment	RS_3_	30.6%	27.0%
		Annealing treatment		28.8%	19.5%
White sweet potato [71]	24%	Heat–moisture treatment		39.3%	63.8%
		Annealing treatment		29.2%	21.7%
Purple sweet potato [71]	25.3%	Heat–moisture treatment		35.4%	39.9%
		Annealing treatment		32.0%	26.5%
Potato starch [72]	11.54%	Microwave-toughening treatment	RS_3_	27.09%	134.7%
Potato starch [60]	64.7%	Microwave irradiation retrogradation treatment	RS_3_	71.5%	10.5%
Potato starch [73]	58.6%	Physically mixed–uncooked treatment	Potato starch–Ser amino complexes	72.1%	23.0%
	4.5%	Physically mixed–cooked treatment	Potato starch–Ser amino complexes	16.1%	257.8%
	27.1%	Heat–moisture treatment–uncooked treatment	Potato starch–Lys amino complexes	70.6%	160.5%
	1.5%	Heat–moisture treatment–cooked treatment	Potato starch–Lys amino complexes	16.2%	980%
	53.7%	Annealing treatment–uncooked treatment	Potato starch–Asp amino complexes	57.2%	6.5%
	5.6%	Annealing treatment–cooked treatment	Potato starch–Asp amino complexes	14%	150%
Purple sweet potato [74]	14.7%	Heat–moisture treatment	Purple sweet Potato starch–citric acid complexes	42.1%	186.4%
			RS_3_	27.2%	85.0%
Potato [75]	22.5%	Heat–moisture treatment	RS_3_	28.5%	26.7%
			Potato starch–citric acid complexes	39.0%	73.3%
Potato starch [76]	12.32%	Heat–moisture treatment	Polyphenol–starch complexes	21.67%	75.9%
Yam starch [74]	21.6%	Heat–moisture treatment	Yam starch–citric acid complexes	46.4%	114.8%
			RS_3_	31.0%	43.5%
Cassava [75]	20.3%	Heat–moisture treatment	RS_3_	26.6%	31.0%
			Cassava starch–citric acid complexes	40.2%	98.0%
Faba bean starch [77]	49.8%	Water bath treatment	RS_4_	61.1%	22.7%
Red kidney beans [78]	21.27%	Enzymatic hydrolysis	RS_3_	31.47%	48.0%
		Autoclaving–enzymatic hydrolysis		42.34%	99.1%
Lotus seeds [79]	35.43%	Autoclaving–cooling treatment	RS_4_	37.68%	6.4%
Lotus seed starch [80]	17.3%	High-hydrostatic pressure treatment	Lotus seed starch–lauric acid complexes	30.3%	75.1%
Lotus seed starch [81]	14.32%	Dynamic high-pressure homogenization treatment	Lotus seed starch–lecithin complexes	54.84%	283.0%
Chestnut starch [60]	73.4%	Microwave irradiation retrogradation treatment	RS_3_	78.0%	6.3%
*Canna edulis* [82]	5.8%	Dual enzymatic hydrolysis and recrystallization treatment	RS_3_	48.23%	731.6%
*Pinellia ternate* starch [83]	63.07%	Autoclaving–cooling treatment–uncooked	RS_3_	27.24%	−56.8%
	21.23%	Autoclaving–cooling treatment–cooked		27.24%	28.3%
*Euryale ferox* starch [84]	10.13%	Autoclaving	RS_3_	18.17%	79.4%
		Enzymolysis autoclaving		18.72%	84.8%
		Dual enzymolysis		17.85%	76.2%
		Purified autoclaving		80.51%	694.8%
		Purified enzymolysis autoclaving		84.00%	729.2%
		Purified dual enzymolysis		88.05%	769.2%
*Pueraria lobata* [85]	24.15%	Debranching and temperature-cycled crystallization treatment	RS_3_	43.93%	81.9%

Abbreviations: Growth, increase in the content of resistant starch after preparation; Native, content of resistant starch in native starch; RS, resistant starch; Yield, content of resistant starch in the product after preparation.

Carbohydrates are an essential component of legumes, but their composition varies across different legume species. Generally, the content of RS and amylose in legumes is higher than that in wheat and potatoes. However, there are few studies on the synthesis of RS from legumes [86]. The proportion of amylose has a significant effect on RS formation. Generally, a higher amylose ratio leads to a higher RS content. The amylose content of pinto beans, chickpeas, and peas is ∼52.4%, 46.5%, and 42.9%–43.7%, respectively [87,88]. Studying the digestibility of legumes, Sandhu et al. [89] found that the RS contents of black gram, chickpea, field pea, lentils, mung bean, and pigeon peas are 60.9%, 54.3%, 58%, 65.2%, 50.3%, and 78.9%, respectively. The amylose content differs due to the different biosynthesis-related enzyme activities of amylose and amylopectin in starch granules [90]. Under ideal conditions, all amylose is dissolved and used to synthesize RS. Then, the final RS content depends on the amylose content in the raw material. Under such ideal conditions, beans synthesize more RS because of their higher amylose content. From this point of view, beans are more suitable for RS synthesis.

In recent years, traditional Chinese medicinal materials have gradually become more well-recognized. Many medicinal foods not only form a part of the daily diet but also play a role in nutraceuticals and health care. Lotus seed was categorized as a dual-purpose resource for food and drugs by the National Health and Planning Commission of China. Lotus seed starch contains 40% amylose and is thus a rich source of amylose [91]. Pueraria starch contains 22.2%–22.9% amylose [92]. Gordon Euryale seeds also have a high content of starch, 37.66% of which is amylose [84]. These Chinese medicinal materials are expected to become important raw materials for RS synthesis.

Even when the same plant material is used, the final yield of RS can differ based on the plant variety. For example, common corn starch produces lower RS yields than waxy or high-amylose corn starch. Trung et al. [71] studied the synthesis of RS_3_ from different varieties of sweet potato and found that with the same synthesis method, purple sweet potatoes provided the highest RS_3_ content. Even the same plant variety can show different starch contents based on its place of origin.

The method of starch extraction can also affect the final yield of RS. For example, commercially purchased starch may be low in amylose because of processing. If starch is directly isolated and extracted from rhizomes, it may contain more RS_1_ and RS_2_. However, these kinds of RS usually have low-thermal stability, are easily destroyed, and cannot be mass-produced, although a high content of RS in the raw material indirectly reflects the high proportion of amylose. As described in an earlier section, the higher the amylose content, the higher is the RS production. The stability of natural RS_2_ is poor. Hence, during RS_3_ synthesis, unstable RS_2_ needs to be converted to heat-resistant RS_3_. However, the proportion of different RSs in a product cannot be accurately measured with current detection methods, and only structural qualitative analysis can be used. Therefore, in some experiments, including the study by Wang et al. [60], the content of RS is not found to change significantly. However, the molecular structure and physicochemical properties of the starch do change, improving the benefits of RS, which can be applied in various fields.

### Synthesis methods

The synthesis method is another important factor affecting the RS yield. Based on their principle, synthesis methods can be roughly divided into 3 categories: physical methods, chemical methods, and enzyme treatment methods [93]. Physical methods have the advantages of low cost, environmental protection, and safety, and they mainly include 2 hydrothermal treatment processes (heat–moisture treatment and annealing treatment) and a variety of nonhydrothermal treatment processes (autoclaving, ultrasonic treatment, microwave treatment, high-hydrostatic pressure treatment, and high-pressure homogenization treatment) [94]. Chemical methods mainly include acid hydrolysis, cross-linking treatment, methylation, and acetylation, and new functional groups are introduced through chemical modification to change the original physical and chemical properties of starch, including its resistance to amylase [95]. In enzymatic hydrolysis, the molecular structure of starch, its molecular size, and its amylose and amylopectin ratio are changed using enzymes [96].

Table 1 lists several studies on the synthesis of RS using different methods. During the review of this literature, we identified 2 major problems. First, although a single synthesis method has been widely studied and has provided reliable results, more and more researchers have turned their attention to the repetition of a single synthesis method or the combination of multiple synthesis methods with the goal of meeting the needs of the modern industry. Second, many studies have combined starch and lipids, proteins, and even some phytochemicals, such as anthocyanins and flavonoids, to generate new substances. It seems that the digestibility of the starch is reduced more substantially when a cross-linking agent is added to the starch solution or when the starch is compounded with other substances. As described in the second section, it is worth exploring whether these complexes can be classified as RS or which type of RS they are if they have the same anti-amylase properties as RS.

Among the various starch complexes, the most well-studied is the starch–lipid complex. The starch–lipid complex has a conformational barrier that hinders the entry of digestive enzymes, and the lipids prevent the hydration of starch granules [97,98]. Similarly, starch can also interact with proteins, which can encase them and create a physical shield, thus reducing contact with digestive enzymes [[99], [100], [101]]. Soy isoflavones and corn starch can form a complex with a novel crystalline structure, which reduces the digestibility of the complex [102]. Amylose, lipids, proteins, and other plant compounds are present in the system during the synthesis of complexes. Theoretically, this process could involve mutual competition when the synthesis conditions are favorable for the formation of RS_3_, RS_4_, and various complexes. Under external factors, such as autoclaving and microwaving, the chain structure of starch cracks, and the internal water is discharged. This leads to the formation of a left-handed hydrophobic helical cavity. On the one hand, a stable double helix structure, RS_3_, may be formed inside the amylose. On the other hand, other ligands—especially lipids with a hydrophilic head and hydrophobic tail—may also enter the cavity to form single helical structures [103]. The mass production of RS_3_ requires a low-temperature aging process. The formation of other complexes will eventually involve a cooling step, resulting in the generation of RS_3_. This review only focuses on the RS generated during the synthesis process and does not deeply analyze the thermal stability, thermodynamic properties, and particle morphology of different products. Further research is needed to examine the changes in the crystal structure and physicochemical properties of the products during the synthesis of novel complexes.

As can be seen from Table 1, a large number of byproducts are generated during RS synthesis. For example, some residual raw materials remain in the reaction system in the form of starch or glucose even after the generated RS is removed. Although the original purpose is achieved (industrializing RS production for use in the food/medical industry), additional substances are also generated. Thus, for energy saving, if we are to carry out the industrial production of RS, we must also consider how to use this “excess waste.” The starch remaining after the synthesis of RS can be converted into other starch products or hydrolyzed into glucose by adding amylase and applied in other areas, such as sugar production.

## Effects of RS

### Meta-analysis

Table 2 [[104], [105], [106], [107], [108], [109], [110], [111], [112], [113], [114], [115], [116]] lists all published meta-analyses of studies examining the effect of RS on human health, including indicators of glucose function, inflammation, lipid metabolism, bowel function, the number of fermentation products, and appetite. In addition to the healthy population, these studies have also been conducted among patients with diabetes, metabolic syndrome (MetS), end-stage renal disease, dyslipidemia, obesity, hyperinsulinemia, colorectal neoplasia, and other related chronic diseases. The types of RS used in these studies were RS_1_, RS_2_, RS_3_, and RS_4_, whereas the forms of intervention included addition in the form of supplements or ingestion through food.

**TABLE 2:** Meta-analyses of studies on resistant starch

Type of RS	Duration and dose	Population	Number of articles and participants	Indicators
RS_2_, RS_3_, RS_4_ [104]	1–4 wk; 22–45 g/d	Healthy adults	9/193	Fecal wet weight, butyrate concentration, fecal PH↑, defecation frequency↔
RS_2_, RS_3_ [105]	2–52 wk; 10–66 g/d	Healthy adults and those with T_2_DM, dyslipidemia, obesity, hyperinsulinemia	14/820	TC, LDL-C↑, triglycerides, HDL-C↔
RS_2_ [106]	1–12 wk; 8–66 g/d	Healthy individuals	20/670	FPG, body weight, HOMA-IR, TC, LDL-C, HDL-C↔, triacylglycerol↑
		Overweight/obesity		Body Weight↔
		MetS		FPG, body weight, HOMA-IR, TC, LDL-C, HDL-C, triacylglycerol↔
		Prediabetes		HbA1c↔
		T_2_DM		Body weight↑, FPG, HbA1c, HOMA-IR, TC, LDL-C, HDL-C, triacylglycerol↔,
RS [107]	3–52 wk	MetS and related disorders	19/1014	FPG, insulin, HbA1c, TC, LDL-C, TNF-ɑ↑ HOMA-IR, triglycerides, HDL-C, CRP, IL-6↔
RS [108]	2–12 wk; 10–45 g/d	Overweight or obese adults	13/428	FPG, insulin, HOMA-S%, HOMA-B%, LDL-C, HbA1c↑
RS [109]	4–52 wk; 8.16–40 g/d	T_2_DM with obesity	14/515	Insulin↑, BMI, FPG, HOMA-S%, HOMA-B%↔
RS, inulin [110]	4 wk to 12 y; 12–30 g/d	Colorectal neoplasia	20/	SCFA, butyrate↔
RS [111]	4–14 wk; 10–45 g/d	Healthy and diseases	13/672	TNF-ɑ, IL-6↑, CRP↔
RS_2_ [112]	4–8.5 wk; 12–16 g/d	ESRD under MHD	5/179	BUN, Scr, IL-6↑, UA, PCS, IS, hs-CRP, albumin, phosphorus↔
RS_2_ [113]	4–12 wk; 10–45 g/d	Renal disease, diabetes, prediabetes, T_2_DM, obesity, and overweight	8/308	TNF-ɑ↑, CRP, IL-6↔
RS_1_, RS_2_ [114]	Acute; 10–45 g	Young healthy adults	4/264	Lower appetite↑
RS [115]	2–12 wk; 5–66 g/d	Healthy individuals and those with overweight and diabetes	19/503	FPG, HOMA-IR↑, HbA1c, insulin, S_I_, AIR, DI, SG, HOMA-β ↔
RS_2_, RS_3_, RS_4_, resistant dextrin [116]	2–12 wk; 7–45 g/d	MetS, T_2_DM, Prediabetes, overweight, ESRD, MHD, PCOS, and DN	16/739	TNF-ɑ, IL-6, TAC↑; CRP, MDA, SOD↔

Abbreviations: ↑: Positive effects; ↔: No significant effects; AIR, acute insulin response; BUN, blood urea nitrogen; CRP, C-reactive protein; DI, disposition index; ESRD, end-stage renal disease; FPG, fasting plasma glucose; HbA1c, glycated hemoglobin; HDL-C, high-density lipoprotein cholesterol; HOMA-IR, Homeostatic Model Assessment of Insulin Resistance; HOMA, homeostatic model assessment; hs-CRP, high sensitivity C-reactive protein; IL-6, interleukin-6; IS, indoxyl sulfate; LDL-C, low-density lipoprotein cholesterol; MetS, metabolic syndrome; MDA, malondialdehyde; MHD, maintenance hemodialysis; PCS, p-cresyl sulfate; RS, resistant starch; SCFA, short-chain fatty acid; Scr, serum creatinine; SG, glucose effectiveness; SI, insulin sensitivity index; SOD, superoxide dismutase; TAC, total antioxidant capacity; TC, total cholesterol; T_2_DM, type 2 diabetes mellitus; TNF-α: tumor necrosis factor-α; UA, uric acid.

Among the 5 articles describing changes in fasting plasma glucose (FPG) after RS treatment, 3 suggested that RS could significantly reduce FPG levels [107,108,115]. A total of 4 studies analyzed fasting insulin levels, 3 of which demonstrated positive effects [[107], [108], [109]]. Four studies tested changes in HbA1c levels, and 3 of them found that RS reduces HbA1c [107,108]. Three articles evaluated insulin resistance (IR) and the function of islet β cells, of which 1 identified statistically significant effects [115]. One meta-analysis suggested that RS can increase insulin sensitivity [108]. Five studies explored changes in inflammatory indicators, of which 4 revealed statistically significant changes in TNF-α [107,111,113,116], and 3 showed a significant reduction in IL-6 [111,112,116] levels following RS intervention. Four meta-analyses examined the effects of RS on blood lipids, and 3 of them identified a significant reduction in LDL cholesterol [105, 107, 108]. Among these 3 articles, 2 showed significant changes in total cholesterol [105,107], and 1 showed a significant change in triglycerides [106]. One study showed that RS_2_ can reduce blood urea nitrogen and serum creatinine concentrations in patients with kidney disease [112], whereas another showed that RS has beneficial effects on gut health in healthy adults [104]. A meta-analysis also examined the acute effects of RS, showing that RS can reduce appetite [114].

These meta-analyses reveal that RS is beneficial for managing chronic diseases and related indicators. However, there are some differences in the results of different studies. These differences can be attributed to the variances in the study population, study period, type of RS used in the study, and quality control during the study.

A study indicated that the consumption of 15–60 g/d of RS is effective in improving glycemia, insulin sensitivity, and satiety in healthy adults [117]. The RS amount needs to be higher in individuals with type 2 diabetes [117]. According to Gao et al. [109], RS supplements can significantly reduce FPG and improve IR when the RS dosage is 30–40 g/d. Xiong et al. [115] showed that RS intake >28 g/d can significantly reduce FBG levels. Yuan et al. [105] suggested that an RS dosage >20 g/d can significantly reduce serum TG concentrations. A meta-analysis of the effect of RS on appetite showed that the influence of RS was greater when the RS dosage was 25 g/d [114].

Thus, our suggestions for future randomized controlled trials are as follows. If the study includes patients with diabetes, a smaller dose of RS may provide a health benefit, but it should not be <10 g/d. When participants are healthy or have other diseases, an RS dosage of ≥20 g/d will be more helpful to observe an improvement. Similarly, in high-dose population studies, it will be necessary to consider the impact on the digestive system when the dose exceeds 45 g/d, as well as the impact of the high dose on participant compliance. Meanwhile, for individuals in whom RS has been a part of the daily diet for a long duration, more long-term clinical evidence is required for the optimal intake dose.

Among the included meta-analyses, only 2 articles found that the duration of treatment has a significant effect. The subgroup analysis conducted by Yuan et al. [105] showed that when the treatment duration was >4 wk, RS had a significant effect on the reduction in total cholesterol and LDL. Vahdat et al. [111] showed that when the duration was <8 wk, RS had a significant effect on the reduction in IL-6 levels. Thus, short-term and low-dose treatment may not produce significant effects. The improvement in chronic disease indicators through dietary intervention is a long-term process. Moreover, different indicators exhibit different change cycles in vivo. For example, Hb1Ac reflects the 3-mo average blood glucose level [118]. If the treatment duration is short and the dose is not high, RS will not produce significant changes in all relevant indicators. Therefore, when deciding the treatment duration, one must consider the time-dependency of the indicator as well.

A study by Gao et al. [109] included a population with diabetes and obesity. This study found that the health effect of RS on IR amelioration in type 2 diabetes mellitus (T_2_DM) with obesity were better than those in T_2_DM alone. Xiong et al. [115] found that the effect of RS on FPG reduction was more obvious in overweight individuals and those with a high risk of diabetes. Wei et al. [116] demonstrated that RS supplementation can significantly reduce serum TNF-α and CRP concentrations in patients with diseases when compared with healthy people. In their study, RS had a more obvious effect on reducing TNF-α and IL-6 in the population with BMI of >25 kg/m^2^. The baseline levels of study end points are higher in participants with diseases, providing greater room for improvement. This makes the effect of RS intervention more obvious. The improvement caused by RS is relatively weak in healthy people with normal baseline indicators. Further, dietary patterns differ among patients with different diseases. For example, participants with diabetes pay more attention to the intake of dietary fiber. During the experiment, if the diet of the control and experimental group is not comparable, confounding factors could eventually interfere with the effect of RS. It is better to measure relevant indicators before the experiment and then also compare the intervention and control groups at the end of the experiment. After study completion, this will not only enable the comparison of differences between the intervention and control groups but also those before and after intervention in the same group. Finally, a comprehensive analysis and discussion can be conducted.

At present, the RS type adopted by most studies is RS_2_. More rigorously designed research and studies on other types of RS are required. Of all meta-analyses, only 1 study showed that RS_2_ could significantly reduce appetite when compared with RS_1_.

By modulating hepatic glycogen structure through the gut–liver axis, promoting the production of starch-degrading enzymes, modulating IR, and remodeling the intestinal barrier, RS could have potential applications in the management of diabetes mellitus and obesity [119,120]. It could also assist in the treatment of chronic kidney disease because it promotes the secretion of GLP-1, regulates T cells, and reduces metabolic endotoxemia and the concentration of nitrogen and nitrogen compounds in the human body [121]. In fact, the health effects of RS discovered so far are attributed to their fermentation and SCFA production in the colon and their modulation of the gut microbiome.

### Functions of SCFA

SCFAs can affect energy metabolism, liver fat metabolism, blood sugar regulation, mineral absorption, and anti-inflammatory mechanisms in humans [122,123]. SCFAs, especially butyrate, also play a key role in the microbiota–brain–gut axis, affecting the development and progression of CNS diseases [124]. As an energy source for enterocytes, butyrate maintains the barrier function of the intestinal epithelium [123]. Butyrate also has the following physiologic effects: upregulation of GLP-1 and peptide YY, which inhibit glucagon secretion, suppress appetite, and increase satiety; inhibition of the inflammatory factor NF-κB; and the inhibition of proinflammatory cytokine production [[125], [126], [127]]. Thus, RS exerts multiple health benefits by producing SCFAs.

### Intestinal flora

Table 3 [[128], [129], [130], [131], [132], [133], [134], [135], [136], [137], [138]] summarizes the changes in the human gut microbiota detected following interventions with RS. The experimental results indicated that RS increases the number of *Bifidobacterium* and *Ruminococcus* bacteria and decreases the number of *Firmicutes*. These microbiota-related changes reduce intestinal permeability and increase anti-inflammatory capacity in the intestines. *Bifidobacterium* and *Ruminococcus* are 2 of the main dominant flora in the human intestinal tract, and they largely affect the function of the entire host microbiome. The increase in *Ruminococcus* is closely related to the increase in intestinal butyrate concentrations [136]. At least 4 studies suggest that RS can increase the amount of *Eubacterium rectale*, which promotes butyrate production [128,129,132,136]. Two population studies confirmed that RS increases the number of *Bacteroidetes*, *Actinobacteria*, and *Lactobacillus* in the gut [128,131]. Laffin et al. [135] suggested that the increase in intestinal *Faecalibacterium* may mediate an important anti-inflammatory effect in patients with chronic kidney disease after RS_2_ intervention. In addition to affecting the number of specific microbes, RS also affects the diversity, richness, and evenness of the gut microbiota [129]. As a prebiotic, RS can also improve the gut microbiota through “cross-feeding” via multiple mechanisms, including the production of SCFAs. Thus, the impact of RS on the intestinal flora has at least 2 aspects. One involves the direct effects of RS on the intestinal environment via the enhancement of beneficial bacteria and inhibition of harmful bacteria. The other involves its indirect influence on the gut microbiota via the promotion of SCFA production.

**TABLE 3:** Population studies examining the effect of resistant starch on the intestinal microbiota

Type	Duration and dose	Population	Microbial groups showing alterations
RS_2_ [128]	3 wk, 33 g/d	13 healthy human subjects	Bacteroidetes, Actinobacteria, *Parabacteroides distasonis*, *Bifidobacterium adolescentis*↑ Firmicutes, Ruminococcaceae, Faecalibacterium↓
RS_4_ [128]			*Ruminococcus bromii, Eubacterium rectale*↑
RS_3_ [129]	3 wk, 25.56 g/d	14 overweight men	*Ruminococcus bromii, Eubacterium rectale*↑
RS_3_ [130]	3 wk, 20 g/d	14 obese men	Ruminococcaceae↑, *Papillibacter cinnamivorans*↓
RS_2_ [131]	4 wk, 8.5 g/d	18 children	Actinobacteria, *Lactobacillus*↑ Firmicutes, *Roseburia*, *Blautia*, *Lachnospiraceae incertae sedis* ↓
RS_2_ [132]	3 wk, 24 g/d	20 young adults	*Bifidobacterium adolescentis*, *Ruminococcus bromii*, *Eubacterium rectale*↑
RS_2_ [133]	12 wk, 21 g/d	84 older and middle-aged adults	*Bifidobacterium*↑
RS_2_ [134]	4 wk, 40 g/d	19 normal weight subjects	Ruminococcaceae_UCG-005↑
RS_2_ [135]	4 wk, 20 g/d; 4 wk, 25 g/d	9 ESRD subjects	*Faecalibacterium*↑
RS_2_ [136]	2 wk, 20–34 g/d	174 healthy young adults	*Ruminococcus bromii*, *Clostridium chartatabidum*, *Eubacterium rectale*↑
RS_2_ [137]	4 wk, 16 g/d	10 HD subjects	*Roseburia*, *Ruminococcus gauvreauii*↑
RS_2_ [138]	1 wk, 14–19 g/d	30 healthy adults	*Ruminococcus*, *Gemmiger*↑

Abbreviations: ↑: Increased; ↓: Decreased; ESRD, end-stage renal disease; HD, hemodialysis; RS, resistant starch.

The impact of RS on intestinal microecology can be divided into different parts. Martínez et al. [128] showed that the response of intestinal microorganisms to RS_2_ is different from their response to RS_4_. RS_4_ induces changes at the phylum level, increasing Actinobacteria and Bacteroidetes and decreasing Firmicutes. RS_2_ does not cause phylum-level changes, but it increases the proportion of *Ruminococcus bromii* and *Eubacterium rectale*. In other words, the range of intestinal microecological changes caused by RS_4_ is greater. Walker et al. [129] found that RS_3_ can increase *R. bromii* and *Eubacterium rectale* in the intestinal flora of overweight men. An in vitro experiment used a pig in vitro fermentation model to study the changes in intestinal microbial composition caused by 3 kinds of RS fermentation [139]. The results showed that the fermentation process and changes in intestinal flora caused by the 3 types of RS were very different. We have reason to believe that there are huge differences between the complex fermentation processes and microbial structure–function effects induced by the different physical structures of RS molecules. This difference exists not only in the different types of RS, but also in the same type of RS obtained from different sources. The difference in intestinal microbial composition caused by the fermentation of different RS substrates could serve as a theoretical basis for the difference in RS function.

Even the same RS can have different effects on the intestinal microecology of different subpopulations. The changes in SCFA concentration and intestinal microflora after the intake of RS were studied in healthy adults in 2 studies [132,136]. Overall, an increasing trend was observed, but individual differences were large. Ordiz et al. [131] revealed no significant difference in intestinal inflammation following RS treatment among children. The effects of RS on the intestinal health of older and middle-aged adults were studied, and a positive effect on the number of *Bifidobacterium* was observed [133]. The 4 abovementioned studies show that RS is promising for improving intestinal ecology in healthy people.

Studies by Laffin et al. [135] and Kemp et al. [137] showed that RS can increase the number of *Faecalibacterium* and *Roseburia*, which are closely related to kidney disease. The studies by Walker et al. [129] and Salonen et al. [130] showed that RS can increase intestinal Ruminoccaceae in overweight and obese participants. The specific effect of RS on the intestinal microflora of individuals with different diseases provides the possibility of targeted disease treatment in the future. The targeted addition of RS that can increase the number of intestinal microbes can be used as a dietary treatment to improve health.

### Other functional properties

In addition to the abovementioned physiologic functions of RS, its other potential effects have also been investigated. Most studies have focused on its application in the prevention and treatment of tumors, especially intestinal tumors. Sasidharan et al. [140] investigated the effect of oral RS supplementation on the prevention of acute radiation proctitis in patients with cervical cancer, but they could not detect any significant positive effect. Malcomson et al. [141] investigated the effect of RS supplementation on crypt cell proliferation in the rectal mucosa of older healthy participants and showed that RS can increase the total number of mitotic cells in the crypts. So et al. [142] evaluated the tolerability of RS_2_ in patients with irritable bowel syndrome and showed that a certain dose of RS was beneficial in this patient group. A randomized controlled study examined the effects of indigestible carbohydrate supplementation on miR-32 expression in colorectal cells and found that miR-32 levels were significantly elevated in the colorectal mucosa of healthy human participants after 50 d of RS+ polydextrose supplementation [143]. In a practical application study, the daily addition of RS-containing potatoes to the diet was not found to adversely affect cardiometabolic risk or gut permeability in US adults with MetS [144]. Studies on prebiotics, such as inulin and fructooligosaccharides, suggest that the anticancer effects of prebiotics may be related to an increase in the amount of butyrate-producing *Eubacterium rectum* [145]. This could explain the effects of RS in preventing colorectal cancer. Irritable bowel syndrome patients show a decrease in intestinal bifidobacterial [146]. RS increases the number of bifidobacteria, thus reducing symptoms. The Mediterranean diet, which includes foods that contain high amounts of RS, consistently tops the list of healthy dietary patterns. Some researchers have advocated adding RS to the nutrition labels of prepackaged foods to help prevent noncommunicable diseases [147]. It is practically significant to add RS to food raw materials as a substitute for refined carbohydrates to control blood sugar levels in patients with diabetes.

## Conclusion and Perspectives

RS has characteristics such as a low water-holding capacity and starch-like texture, but it has the physiologic functions of dietary fiber. RS can be added to staple foods, such as bread, noodles, cakes, etc., to improve the taste of food and its nutritional value. In the future, RS can be used as a food additive to develop foods with a low GI and low-caloric value [148]. It can also be used as an emulsifier and thickener to improve the sensory properties of food [149]. Modified starch has promising applications in biodegradable food packaging and biologic films [150]. RS can not only support intestinal fermentation to increase the number of probiotics, but it can also be used as a carrier of probiotics due to its nondigestible characteristics. Thus, it can help probiotics avoid decomposition and destruction in the digestive system and accumulate in the colon. The starch–lipid complex has a unique spiral cavity structure that can be used as a carrier for targeted drug delivery. For example, starch–lipid–protein complexes are used as carriers of chemotherapeutic drugs [151].

The European Food Safety Authority declared that RS is beneficial for postprandial blood glucose levels [152]. The FDA has agreed that high straight-chain corn RS can reduce the risk of T_2_DM [153]. The FDA has recognized that some types of RS, especially high-amylose starch containing RS_2_, can be considered as dietary fiber in nutritional ingredient and supplement ingredient labels. We believe that the RS content should be included in nutrition labels to allow consumers to make more informed choices. This could serve as a strategy to prevent chronic diseases, such as diabetes, obesity, dyslipidemia, and colorectal cancer.

All types of clinical research have the same ultimate goal, ie, improving the lives of human beings. Research on blood sugar-related indicators shows that RS can be added to food to control blood sugar levels and improve quality of life in patients with diabetes. Similarly, studies on blood lipids and inflammation indicate that RS can act as an adjuvant for the management of MetS and inflammation-related diseases. From a public nutrition perspective, the intake of reasonable doses of RS as part of the daily diet can provide health benefits. From a clinical nutrition perspective, RS can help in disease prevention and treatment through diet fortification. The selection of appropriate raw materials and synthesis methods, and the study of digestive processes and physiologic functions of RS are key aspects of research. For practical application, a series of problems need to be solved, including the dosage and type of RS, targeted user population, method of administration, and interaction with other foods. This is a long road, and more researchers should devote their efforts toward addressing these issues.

## Acknowledgments

The authors’ responsibilities were as follows – YW: conceptualization of the review; ZW, SW: writing; QX, QK, FL, LL, YX: responsible for the final content; and all authors: read and approved the manuscript.

### Author disclosures

The authors report no conflict of interest.

### Funding

Supported by the Anhui Provincial Natural Science Foundation (grant number: 2208085MH244) and the Major Difficult Diseases Key Project of Integrated Chinese and Western Medicine of Administration of Traditional Chinese Medicine of Anhui Province (2021zdynjb10).
